# Multiscale Architecture and Mechanics of the Cell Nucleus: Implications for Disease, Bioengineering and Nanomedicine

**DOI:** 10.1002/advs.75470

**Published:** 2026-05-07

**Authors:** Xinran Liu, Xin Chen, Qingyang Zhao, Weikang Tan, Lingrui Zhu, Kin Liao, Vincent Chan, Nicolae Goga, Winfred O. Larkotey, Yang Li, Yinghui Shi, Wenya Shu, Lin Wang, Weiqing Wang, Tian Jian Lu, Yulong Han

**Affiliations:** ^1^ State Key Laboratory of Mechanics and Control for Mechanical Structures Nanjing University of Aeronautics and Astronautics Nanjing China; ^2^ Advanced Digital and Additive Manufacturing Center Khalifa University of Science and Technology Abu Dhabi UAE; ^3^ Molecular Dynamics Group University of Groningen Groningen The Netherlands; ^4^ Department of Computing Sciences and Engineering Valley View University Accra Ghana; ^5^ Engineering Research Center of Personalized Anti‐Aging Health Product Development and Transformation of Universities of Shaanxi Province and College of Medicine Xi'an International University Xi'an China; ^6^ Department of Otolaryngology Peking Union Medical College Hospital Chinese Academy of Medical Science and Peking Union Medical College Beijing China

## Abstract

The cell nucleus serves as the central hub of genetic information and a dynamic, multiscale mechanosensor that regulates critical cellular behaviors, such as gene expression and cell migration. Functioning as a complex soft matter system, the nucleus comprises a hierarchical architecture spanning from nanoscale DNA and nucleosomes to microscale chromosome territories and the nuclear envelope. This multiscale structural organization endows the nucleus with scale‐dependent mechanical properties—such as stiffness and viscoelasticity—that are fundamental to cellular mechanotransduction and the strict regulation of nuclear entry for therapeutic nanoparticles. This review emphasizes the recent advances in understanding the multiscale architecture and mechanics of the cell nucleus. We compare experimental technologies for probing nuclear mechanics, alongside emerging multiscale theoretical models that transform these qualitative observations into quantitative, predictive frameworks for mechanobiology and nanomedicine. Finally, we highlight the translational implications of nuclear mechanics on nuclear‐targeted nanomedicine, mechanodiagnosis, and mechanotherapy.

## Graphical abstract

Nuclear mechanical properties are inherently scale‐dependent, arising from a hierarchical architecture that spans DNA, chromatin, the nuclear envelope, and condensates. Experimental techniques and theoretical models are integrated into a cohesive multiscale framework linking nanoscale structural features to organelle‐level mechanical behavior. These insights open new directions in mechanodiagnostics, mechanotherapy, and nucleus‐targeting nanomedicine.

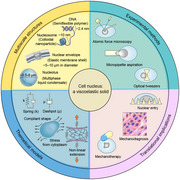

## 1 Introduction

The cell nucleus is the largest and stiffest organelle within eukaryotic cells, functioning as both a genetic repository and a central mechanosensory hub [1, 2]. It endures physical forces that induce structural deformations across multiple length scales. These deformations actively regulate essential cellular processes, from chromatin accessibility and gene expression to cell migration through confined environments [1, 3, 4]. Nuclear deformation can also compromise nuclear envelope integrity, causing rupture‐associated DNA damage that links mechanical perturbation directly to genomic instability [5, 6, 7, 8]. Beyond mechanobiology, the mechanical properties of the nuclear envelope regulate nucleocytoplasmic transport and determine the efficiency of nucleus‐targeted drug delivery. Understanding nuclear mechanics thus opens new avenues in mechanomedicine, with broad implications for disease diagnosis and therapy, and informs the rational design of nanomedicine strategies where the nuclear envelope acts as a physical gatekeeper.

The nuclear mechanics originates from a highly integrated multiscale architecture [9]. At the nanoscale, DNA and chromatin form a tunable viscoelastic scaffold that regulates transcription and DNA repair [1]. At the mesoscale, phase separation concentrates intrinsically disordered proteins into membraneless condensates whose interfacial and viscoelastic properties organize subnuclear compartments. At the microscale, the nuclear envelope and lamina transmit cytoskeletal forces to control nuclear shape and stability. When this cross‐scale mechanical coupling is disrupted—as in confined 3D microenvironments that persistently remodel nuclear structure and function [10, 11, 12, 13]—cellular homeostasis fails, driving the pathogenesis of conditions ranging from rare laminopathies to various cancers [14].

Characterizing these properties across length scales requires a diverse experimental toolkit. Contact‐based platforms such as atomic force microscopy, tweezers, and micropipette aspiration apply calibrated forces to quantify stiffness, viscoelasticity, and force–extension behavior at defined resolutions. Passive imaging modalities, including particle tracking and Brillouin microscopy, complement these approaches by mapping intranuclear rheology without external perturbation. To convert these measurements into mechanistic understanding, researchers employ multiscale theoretical models [15]. Statistical polymer frameworks describe the flexibility and mechanics of DNA and chromatin fibers [16, 17], while continuum and poroelastic formulations capture whole‐nucleus properties such as creep compliance and fluid redistribution under load [18]. Together, these experimental and computational tools provide the quantitative basis for linking nanoscale structural alterations to changes in organelle‐level mechanical behavior.

Exploring nuclear mechanics offers promising translational opportunities in mechanomedicine and nuclear‐targeted nanomedicine. In mechanomedicine, quantitative mechanical parameters now serve as label‐free biomarkers for AI‐driven mechanotyping of disease [19]. Moreover, regulating nuclear mechanics emerges as a promising mechanotherapeutic strategy for cancer and aging [20, 21, 22, 23]. In nanomedicine, nuclear mechanics is opening innovative pathways for nanoparticle design. Rather than relying solely on biochemical targeting, emerging strategies now leverage mechanical principles to engineer nanocarriers whose size, shape, stiffness, and deformability are precisely optimized to efficiently overcome the physical constraints imposed by the nuclear envelope [24, 25]. Thus, elucidating nuclear mechanical constraints provides a robust, quantitative basis for advancing mechanics‐guided precision medicine.

In this review, we establish a cohesive multiscale framework for nuclear mechanics. We first outline the hierarchical architecture of the cell nucleus that governs its mechanical properties. Next, we synthesize the complementary experimental toolkits and theoretical models used to quantify these properties. Finally, we discuss the translational impact of nuclear mechanics in mechanomedicine and nanomedicine.

## 2 Multiscale Structures of the Cell Nucleus

**FIGURE 1:**
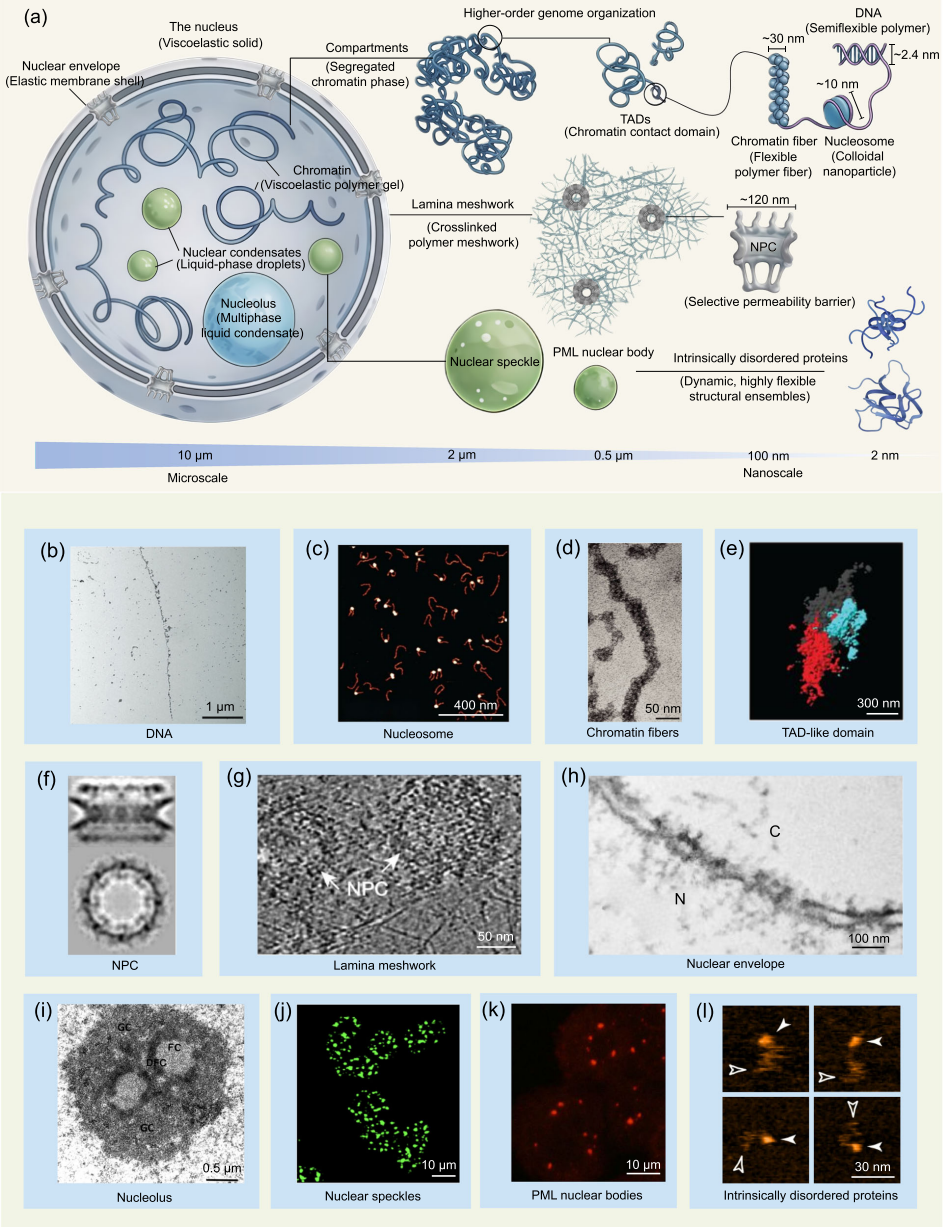
Multiscale nuclear structures. (a) Overview of nuclear structures from the nanoscale to the microscale and their material properties. (b) Transmission electron microscopy image of HeLa genomic DNA at high magnification. Adapted with permission [204]. Copyright 2024, John Wiley and Sons. (c) AFM image of nucleosomes with flanking DNA arms. Adapted with permission [205]. Copyright 2019, Journal of Visualized Experiments. (d) Electron micrograph of a 30 nm chromatin fiber. Adapted with permission [206]. Copyright 2003, Springer Nature. (e) A TAD‐like chromatin domain visualized by single‐cell super‐resolution chromatin tracing. Adapted with permission [207]. Copyright 2018, AAAS. (f) Side and top view projections of the NPC revealing its spoke ring organization. Adapted with permission [208]. Copyright 2012, Elsevier. (g) Electron tomography of the lamina meshwork in the mammalian cell nucleus, with NPCs indicated by arrows. Adapted with permission [39]. Copyright 2017, Springer Nature. (h) Electron micrograph of the nuclear envelope, with the nucleoplasmic side labeled N and the cytoplasmic side labeled C. Adapted with permission [209]. Copyright 2008, Elsevier.(i) Electron micrograph of the nucleolus showing the FC, DFC, and GC. Adapted with permission [210]. Copyright 2015, Springer Nature. (j) Nuclear speckles in HeLa cells. Adapted from [211] under CC BY 4.0. (k) PML nuclear bodies in THP‐1 cell nuclei. Adapted with permission [212]. Copyright 2022, Springer Nature. (l) High‐speed AFM images of IDPs (open arrowheads) connected to folded globular protein domains (closed arrowheads). Adapted with permission [213]. Copyright 2020, Springer Nature. Abbreviations: AFM, atomic force microscopy; TAD, topologically associating domain; NPC, nuclear pore complex; FC, fibrillar center; DFC, dense fibrillar component; GC, granular component; PML, promyelocytic leukemia; IDPs, intrinsically disordered proteins.

The nucleus consists of three primary structural components: chromatin, nuclear envelope, and nuclear condensates (Figure 1a). Chromatin acts as a tunable polymer scaffold whose hierarchical organization underpins genome organization and transcriptional regulation. The nuclear envelope serves as a selectively permeable mechanical shell, defining the nucleocytoplasmic boundary to ensure stability and mediate molecular transport. Nuclear condensates further compartmentalize essential biochemical processes, such as RNA processing, into discrete subnuclear domains. Collectively, these components establish a complex multiscale architecture that spans from nanoscale DNA dynamics to microscale cell mechanics (Table 1), orchestrating gene expression and organismal homeostasis. Deciphering how mechanical forces propagate across these scales is critical for elucidating nuclear regulation in health and developing mechanotargeted therapies for disease.

**TABLE 1:** Mechanical properties of nuclear structures measured by diverse experimental methods.

Multiscale nuclear structures		Mechanical properties and response descriptors	Measurement results	Methods	Refs.
chromatin	double‐stranded DNA	overstretching force	∼65 pN	OT	[183]
		stretch modulus	∼1100 pN	OT	[184]
		bending persistence length	∼50 nm	MT	[85]
			∼53 nm	OT	[184]
			∼22–96 nm	AFM	[185]
		torsional persistence length (high forces)	∼100 nm	MT	[86]
		torsional persistence length (low forces)	∼40 nm	MT	[86]
	nucleosome	unwrapping force	3–6 pN	MT	[84, 186]
	chromatin fiber	stretch modulus	∼5 pN	OT	[187]
		bending persistence length	∼30 nm	OT	[187]
		twist persistence length	∼5 nm	MT	[83]
	chromosome	young's modulus	∼0.39 kPa	AFM	[188]
		stretch modulus (anti‐histone antibody attachment)	∼30 pN	stretching methods	[189]
		stretch modulus (anti‐SMC antibody attachment)	∼180 pN	stretching methods	[189]
		stretch modulus	∼120 pN	micropipette aspiration	[190]
		relaxation time (mitotic)	∼2 s	micropipette aspiration, stretching methods	[191, 192]
		relaxation time (interphase)	∼20 s	stretching methods	[192]
nuclear envelope	lamin A filament	elastic limit force	∼500 pN	AFM	[139]
		strain‐stiffening threshold	∼2 nN	AFM	[139]
	nuclear pore complex basket structure	local stiffness	2–5 pN/nm	AFM	[193]
	nuclear pore complex scaffold ring	local stiffness	4–8 pN/nm	AFM	[193]
	lamina	network elastic modulus	∼25 mN/m	micropipette aspiration	[94]
		stretching stiffness	∼5 mN/m	micropipette aspiration	[194]
nuclear condensates	nucleolar dense fibrillar component	elastic modulus	2.6 ± 0.7 Pa	micropipette aspiration	[99]
	nucleolus	longitudinal modulus	2.487 ± 0.005 GPa	Brillouin microscopy	[113]
the whole nucleus		yield stress	50–150 pN	particle tracking+MT	[195]
		storage modulus	8 ± 3 Pa	particle tracking	[105]
		elastic modulus	0.28–9.1 kPa	AFM	[196, 197]
			1–5.7 kPa	micropipette aspiration	[172]
		elastic modulus (in cell)	∼5 kPa	compression methods	[74]
		elastic modulus (isolated)	∼8 kPa	compression methods	[74]
		apparent Poisson's ratio	0.40–1.21	compression methods	[198]
		nucleus: cytoplasm stiffness ratio	∼1.4	compression methods	[75]
		instantaneous viscosity	∼5 kPa·s	micropipette aspiration	[172]
		equilibrium viscosity	21 ± 2 kPa·s	micropipette aspiration	[172]
		longitudinal modulus (isolated)	2.42 ± 0.03 GPa	AFM	[199]
		longitudinal modulus (in intact cell)	2.61 ± 0.04 GPa	Brillouin microscopy	[199]
		apparent Young's modulus	0.76–8 kPa	micropipette aspiration, AFM, MT	[87, 200]
		viscosity	6.31–6.98 kPa·s	MT	[87]
		relaxation time (suspension)	6.08 ± 1.1 s	OT	[80]
		relaxation time (confined)	4.00 ± 0.6 s	OT	[80]

### 2.1 Chromatin Organization across Scales

Chromatin functions as a sophisticated, hierarchical biopolymer system spanning multiple length scales. At the nanoscale, DNA is tightly wrapped around histone octamers, forming nucleosomes that eventually assemble into flexible chromatin fibers. At the mesoscale, these chromatin fibers organize into topologically associating domains (TADs), which further cluster into larger chromatin compartments. At the microscale, whole chromosomes occupy discrete spatial volumes known as chromosome territories, effectively defining the global rheology of the nucleus. This multiscale architecture does more than efficiently package the genome; it establishes a mechanically tunable scaffold where local stiffness variations directly regulate gene expression, DNA repair, and nuclear stability. While nuclear structures at different scales possess distinct physical properties (e.g., from the persistence length of DNA to the viscoelasticity of chromatin territories), they remain dynamically coupled. Unraveling these multiscale mechanically coupled interactions is essential for elucidating how physical forces drive cell fate and for identifying novel mechanical biomarkers of disease.

#### 2.1.1 DNA

At nanoscale, DNA functions mechanically as a semiflexible biopolymer that forms the structural backbone of chromatin (Figure 1a,b). As a double‐helical molecule of approximately 2 nm in diameter [26], DNA exhibits polymer mechanics characterized by three key parameters: stretch modulus, bending persistence length, and torsional persistence length (Table 1, double‐stranded DNA). This mechanical behavior is characteristically described by the worm‐like chain framework. Consequently, sequence‐dependent variations in DNA stiffness and bendability across the genome establish a fundamental “mechanical code” that dictates local physical properties at the molecular level.

Rather than acting purely as a passive genetic repository, the mechanical deformability of DNA directly governs its biological function. Its inherent flexibility permits the precise bending, twisting, and looping required for transcription factor assembly and long‐range genomic interactions [27]. Furthermore, when subjected to applied tension or torsional stress, DNA undergoes force‐driven structural transitions—such as overwinding, unwinding, and supercoiling—that physically modulate local chromatin accessibility and compaction [28]. Ultimately, the nanoscale mechanical response of DNA to these physical forces provides the biophysical foundation upon which higher‐order chromatin architecture and larger‐scale nuclear mechanical behaviors emerge.

#### 2.1.2 Nucleosomes and Chromatin Fibers

Hierarchically, DNA wraps around a rigid histone octamer core to form nucleosomes [29], which is approximately 10 nm in diameter (Figure 1a,c). This nucleosome assembly is mechanically dynamic, as force can induce nucleosome unwrapping at the piconewton scale. The nucleosome arrays then condense into higher‐order fibers. While traditionally represented as uniform 30 nm fibers (Figure 1a,d), recent evidence reveals that in vivo chromatin primarily adopts irregular and dynamic structures [30], resulting in mechanical heterogeneity and adaptive responses. At the fiber level, chromatin mechanics can be characterized by the stretch modulus, bending persistence length and twist persistence length (Table 1, chromatin fiber). Furthermore, chromatin behaves as a stimuli‐responsive polyelectrolyte [31]. Changes in ionic strength modulate electrostatic screening between DNA segments, thereby altering chromatin compaction.

The adaptable conformational dynamics of chromatin fibers acts as a mechanically tunable viscoelastic polymer system within the nucleus, through which biochemical and ionic conditions modulate chromatin folding states to influence gene regulation [32]. For example, chromatin compaction can protect genomic DNA from radiation‐induced damage and chemical attack, thereby helping to maintain genome integrity [33]. Regions of high nucleosome density assemble into stiff, compact fibers, which are generally associated with transcriptional silencing, whereas lower‐density regions retain high flexibility, facilitating the bending dynamics required for gene transcription and DNA repair [34]. Collectively, these findings demonstrate that chromatin mechanics is not simply a passive consequence of genome packaging, but an active determinant of nuclear function.

#### 2.1.3 Higher‐Order Genome Organization

Beyond the fiber level, the genome self‐organizes into a hierarchical metamaterial composed of TADs, chromatin compartments, and chromosomes. TADs are intermediate‐scale chromatin structures (∼0.1–2 megabases in size) characterized by frequent internal chromatin interactions and discrete boundaries (Figure 1a,e). On a global scale, the genome undergoes microphase separation into transcriptionally active euchromatic and inactive heterochromatic compartments. In interphase nuclei, chromosomes occupy discrete regions known as chromosome territories [35], while during mitosis they achieve their highest level of compaction, forming rod‐shaped structures with distinct mechanical properties, including the Young's modulus, stretch modulus and relaxation time (Table 1, chromosome).

The mechanics of higher‐order genome organization influence transcription, genome stability, and developmental processes. TADs preserve domain integrity by isolating local stiffness variations and chromatin remodeling events from neighboring genomic regions, thereby enabling precise, localized mechanotransduction responses to environmental stress. At the compartment level, euchromatic compartments form an open, solvated polymer network that maximizes transcriptional responsiveness, while heterochromatic compartments collapse into dense, hydrophobic‐like clusters that impart structural rigidity at the nuclear periphery. Furthermore, mitotic chromosome stiffness and stability are required for faithful chromosome segregation [36], whereas segregation defects can contribute to developmental abnormalities and cancer [37].

### 2.2 Nuclear Envelope

The nuclear envelope functions as a hierarchical nanocomposite interface bridging the nanoscale and microscale processes. Its architecture integrates nuclear pore complexes (NPCs), the nuclear lamina, and nuclear membranes into a cohesive system. NPCs, large protein assemblies approximately 120 nm in diameter, are embedded within the nuclear membranes, selectively regulating molecular transport between the nucleus and cytoplasm. Positioned at mesoscale intervals of several hundred nanometers (e.g., median spacing of ∼560 nm in HeLa cells [38]), NPCs serve as discrete anchoring nodes that structurally support the nuclear lamina meshwork. The nuclear lamina is a dense, fibrous protein meshwork, primarily composed of lamins, which provides mechanical support and anchors peripheral chromatin domains [39, 40]. Structurally coupled to the nuclear membranes and anchored at NPCs, the lamina imparts rigidity, elasticity, and overall mechanical stability to the nucleus. Together, these integrated components form the global nuclear envelope, which typically spans approximately 5–10 µm in diameter. This nested, multiscale organization ensures mechanical integrity, spatial compartmentalization, and precise functional control of nuclear–cytoplasmic communication, which is critical for cellular homeostasis and function.

#### 2.2.1 Nuclear Pore Complexes

NPCs function as sophisticated biological nanopores embedded within the nuclear envelope, acting as the sole gateways for selective, bidirectional molecular transport. Passive diffusion through the NPC is limited to small molecules, whereas transport of molecules larger than ∼5 nm requires binding to nuclear transport receptors. As massive supramolecular assemblies, each NPC is self‐assembled from approximately 30 distinct nucleoporins (Nups) arranged with octagonal symmetry around a central channel [41] (Figure 1a,f).

NPCs exhibit dynamic structural adaptability and functional plasticity [42], which link changes in nuclear envelope tension to transport‐dependent nuclear responses [10], with downstream consequences for genome regulation [43]. Scaffold Nups confer mechanical integrity and stability to the nuclear envelope, whereas peripheral Nups are implicated in interactions at the nuclear periphery [44]. Alterations in nuclear pore complex components and in nucleocytoplasmic transport are associated with human disease, including neurodegenerative disorders [45] and cancers [46], underscoring the importance of nuclear pore integrity and transport homeostasis.

#### 2.2.2 The Lamina Meshwork

The nuclear lamina is a dense fibrillar meshwork composed mainly of intermediate filament proteins known as A‐type (lamins A and C) and B‐type (lamins B1 and B2) lamins, located immediately beneath the inner nuclear membrane (Figure 1a,g). These lamins assemble into a robust, cross‐linked network of filaments approximately 10 nm in diameter, which is critical for maintaining nuclear morphology, mechanical integrity, and anchoring peripheral chromatin domains. In mechanical models, the lamina is commonly represented as a thin elastic or viscoelastic shell, while the biopolymeric shell model specifically treats it as a cross‐linked polymer mesh. At the meshwork level, lamina mechanics can be characterized by the network elastic modulus and stretching stiffness (Table 1, lamina). Specifically, A‐type lamins, predominantly expressed in differentiated cells, confer nuclear stiffness and regulate nuclear shape. In contrast, B‐type lamins, which are ubiquitously expressed across somatic cells, provide essential structural support for nuclear envelope integrity [47].

Moreover, the nuclear lamina significantly modulates nuclear mechanical stability and viscoelastic responses, enabling dynamic adaptation to changes in cytoskeletal tension and extracellular matrix stiffness. This mechanical responsiveness critically influences processes such as stem cell differentiation, collective cell migration, and cellular responses to mechanical stresses [48, 49]. Dysfunction or mutations in lamins or lamin‐associated proteins compromise nuclear mechanics and disrupt chromatin organization, leading to diverse diseases known as laminopathies, including Emery–Dreifuss muscular dystrophy, familial partial lipodystrophy, and Hutchinson–Gilford progeria syndrome [47]. Thus, the nuclear lamina acts as a central mechanosensitive element, crucially bridging nuclear structural integrity and cellular mechanotransduction under both physiological and pathological conditions.

#### 2.2.3 The Nuclear Envelope

The nuclear envelope consists of three key structural components: NPCs, the nuclear lamina, and the nuclear membranes (Figure 1a,h). The nuclear membranes form a double lipid bilayer structure surrounding the nucleus, acting as a physical barrier between nuclear contents and the cytoplasm. As a continuous membrane system, the nuclear envelope therefore functions as an elastic shell that supports the mechanical stability of the nucleus. The nuclear envelope also acts as a dynamic mechanosensor, primarily through the linker of nucleoskeleton and cytoskeleton (LINC) complex, which physically couples the cytoskeletal network in the cytoplasm to the nuclear lamina beneath the inner nuclear membrane.

The nuclear envelope couples mechanical cues to nuclear responses such as DNA damage. Through the LINC complex, the nucleus can adaptively respond to physical stimuli encountered during essential cellular processes, including migration, differentiation, and development [49]. Conversely, excessive or pathological mechanical stresses can disrupt the nuclear envelope integrity, leading to chromatin herniation, DNA damage, and activation of cellular stress response pathways—events closely associated with aging‐related diseases and immune dysfunction [1, 50].

### 2.3 Nuclear Condensates

Nuclear condensates represent hierarchical assemblies generated through liquid–liquid phase separation, including the nucleolus (Figure 1a,i), nuclear speckles (Figure 1a,j), and promyelocytic leukemia (PML) nuclear bodies (Figure 1a,k). At the molecular level, the amino acid sequence of intrinsically disordered proteins (IDPs) affects the mechanics of nuclear condensates through various molecular interaction (e.g., π–π interaction and electrostatic interactions). At organelle level, the surface tension and viscoelastic properties of nuclear condensates critically maintain the organelle architectures and biological function. This sequence‐dependent mechanics underpins condensate structure and function, establishing a multiscale connection between the molecular and organelle levels.

#### 2.3.1 Intrinsically Disordered Proteins

As the primary driver of nuclear condensate formation, IDPs lack a fixed or ordered 3D structure, thereby enabling extensive multivalent interactions among their residues (Figure 1a,l). IDPs contain diverse amino acid residues, such as aromatic residues and charged residues. These two types of residues mediate the π–π interaction and electrostatic interactions respectively, which are crucial for the microscale mechanics of nuclear condensate. Aromatic residues, including tryptophan, tyrosine, and phenylalanine contain an aromatic ring structure. The delocalized π‐electron systems in the aromatic ring structures enable the formation of a variety of strong molecular interactions including π–π stacking and cation–π interactions [51, 52, 53]. In addition to aromatic residues, cationic residues and anionic residues carry formal charges and engage in long‐range electrostatic interactions. These interactions can be either attractive or repulsive over nanometer‐scale distances, thereby strongly influencing condensate mechanics [54]. Collectively, these aromatic and charged residues not only serve as the fundamental building blocks of IDPs, but also provide the molecular basis for determining condensate mechanical properties.

These sequence‐specific interactions regulate the microscale mechanics of nuclear condensates. On the one hand, the types of residues affect the strength of the molecular interaction, thus the resulting mechanical properties of the condensates. Recent microrheology measurements on heterogeneous nuclear ribonucleoprotein condensates showed that freshly formed condensates behave as viscoelastic Maxwell fluids, with storage modulus (G′) and loss modulus (G″) all depending strongly on types of residues [55, 56]. Specifically, stronger aromatic residues increase both elastic and viscous moduli and shift the condensate toward more elastic behavior over longer timescales; condensates formed with tryptophan‐rich residues are mechanically stronger than those enriched in tyrosine. On the other hand, charge patterning provides an additional route for tuning condensate mechanics through regulating the strength of electrostatic interaction and the lifetime of transient intermolecular contacts [57, 58]. Computational analyses of charge‐rich disordered proteins showed that increasing charge segregation slows condensate dynamics, decreases molecular diffusion, and monotonically increases both zero‐shear viscosity and surface tension [58]. Together, these findings show that IDP sequence determines not only whether condensates form, but also their viscoelasticity, interfacial mechanics, and aging behavior.

#### 2.3.2 Nucleolus

The nucleolus is a multilayered organelle in the cell nucleus whose primary function is to produce and assemble the ribosomes [59]. The multilayered structure, formed through liquid–liquid phase separation, is maintained by the distinct mechanical properties (e.g., surface tension) of the constituent condensates. The nucleolus comprises three immiscible condensate layers: the innermost fibrillar center (FC), the intermediate dense fibrillar component (DFC), and the outer granular component (GC) [60, 61]. This hierarchical architecture is governed by a gradient of surface tensions that emerges from differences in molecular composition. In particular, the granular component, which is enriched in nucleophosmin, exhibits the lowest surface tension and therefore forms the outer shell, whereas the dense fibrillar component, enriched in fibrillarin, has a higher surface tension and is consequently positioned more internally [59, 62]. Thus, interfacial tension differences among nucleolar layers play a central role in establishing and maintaining nucleolar spatial organization.

The mechanical properties of condensates not only determine the multilayered structure of the nucleolus but also regulate the synthesis of ribosomes. In the synthesis of ribosomes, the nucleolus exhibits a radial viscoelastic gradient from an elastic core to a liquid‐like shell. This gradient constrains nascent rRNA mobility in the core for transcription and facilitates the release of assembled preribosomal particles in the shell [62]. Conversely, aberrations in nucleolar mechanics are tightly linked to pathological dysfunction of ribosome production. In C9orf72‐linked amyotrophic lateral sclerosis, proline–arginine dipeptide repeats infiltrate the nucleolus, markedly decreasing nucleolar protein nucleophosmin and entrapping rRNA in static condensates. The resulting loss of nucleolar fluidity directly impairs rRNA processing, thereby disrupting ribosome biogenesis [63]. Thus, Nucleolus mechanics is not merely a physical attribute but also shapes nucleolar structure and its function.

## 3 Experimental Methods to Characterize Nuclear Mechanics

**FIGURE 2:**
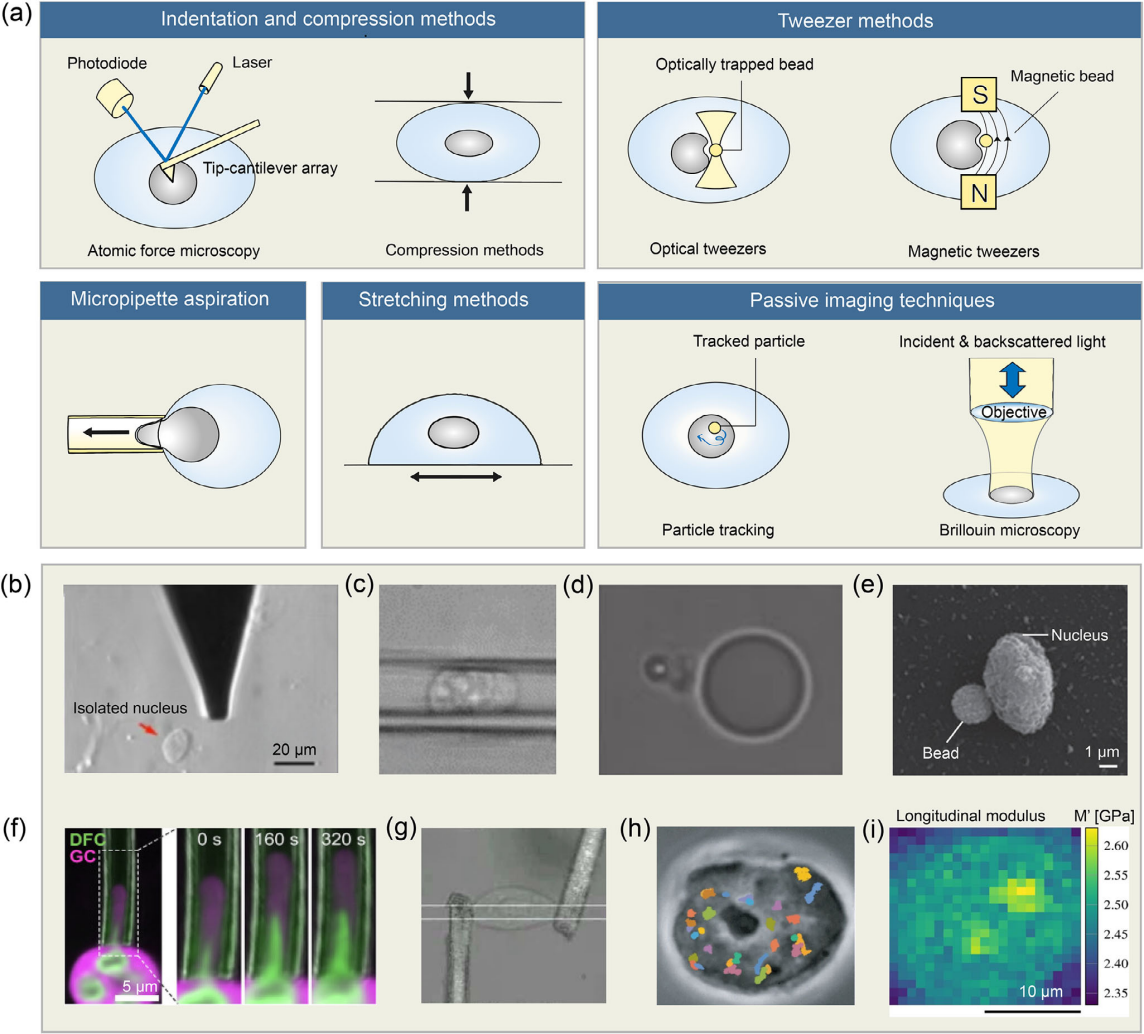
Experimental approaches to measure the physical properties of the cell nucleus. (a) Overview of experimental approaches used to measure the physical properties of nuclear structures. (b) AFM indentation of an isolated nucleus. Adapted with permission [214]. Copyright 2014, American Chemical Society. (c) Compression of an isolated nucleus between a rigid microplate and a flexible microplate. Adapted with permission [74]. Copyright 2002, Elsevier. (d) Transmitted light image of an isolated nucleus attached to a poly‐ornithine‐coated bead in an OT assay. Adapted from [81] under CC BY 4.0. (e) Scanning electron micrograph of a magnetic bead attached to an isolated nucleus in a MT assay. Adapted with permission [215]. Copyright 2014, Springer Nature. (f) Micropipette aspiration of GC and DFC of a nucleolus. Adapted from [99] under CC BY 4.0. (g) Microstretching of a spherical endothelial cell using cantilevers to probe deformation of the nucleus inside the cell. Adapted with permission [101]. Copyright 2007, Mathematical Sciences Publishers. (h) Particle tracking of isolated Jurkat nuclei. Adapted from [105] under CC BY 4.0. (i) Brillouin microscopy map showing a higher longitudinal modulus in the nucleoplasm than in the surrounding cytoplasm of a HeLa cell. Adapted from [113] under CC BY 4.0. Abbreviations: AFM, atomic force microscopy; OT, optical tweezers; MT, magnetic tweezers; GC, granular component; DFC, dense fibrillar component.

Given that nuclear mechanics inherently spans length scales from single‐molecule DNA dynamics to whole‐organelle deformation, no single technique can capture its full physical complexity. Instead, a diverse nanomechanical toolkit has been established to probe these properties at specific resolutions (Figure 2a). These experimental platforms broadly fall into five categories based on their mode of force application and scale of interrogation: (1) indentation and compression methods such as atomic force microscopy (AFM); (2) tweezer methods including optical tweezers (OT) and magnetic tweezers (MT); (3) micropipette aspiration; (4) stretching methods; and (5) passive imaging modalities including particle tracking and Brillouin microscopy. Table 2 synthesizes these methodologies, contrasting their force ranges and spatiotemporal resolutions to guide the selection of the appropriate tool for specific nuclear structural modules.

**TABLE 2:** Experimental approaches to measure the physical properties of the nucleus.

Method class		Force range	Spatial resolution	Advantages	Limitations
Indentation and compression methods	AFM	∼10 pN–100 nN [64]	Sub‐nanometer (0.5–1 nm) [187]	High force and spatial resolution; Capable of identifying local heterogeneity	Low throughput
	Compression methods	∼1 nN–1 µN [201]	N/A	Easy to implement; Simple parallel‐plate geometry creates a uniform, uniaxial stress field	Low spatial and force resolution; Complex chemical treatments of the plates;
Tweezer methods	OT	∼0.1–100 pN [65]	Sub‐nanometer (0.1–2 nm) [65]	High force and spatial resolution; 3D measurement; Excellent for probing high‐frequency mechanical responses.	Low throughput; Local heating and photodamage; Force limit is too low to induce global deformation of the stiff nucleus
	MT	∼10 fN–1 nN [82]	Nanometer‐scale (5–10 nm) [65]	Capable of probing torque (ideal for DNA supercoiling and chromatin fibers); No sample heating or photodamage; Supports high‐throughput measurements by tracking multiple beads simultaneously	Low spatial precision; Require bead functionalization; No 3D manipulation in permanent‐magnet configurations
Micropipette aspiration		∼10 pN–1 nN [93]	Nanometer‐scale (±25 nm) [93]	Capable of probing nuclear envelope mechanics; Capable of imposing large deformations	Low throughput; Requires skilled operation; Dependent on variable, custom‐pulled pipettes; Analysis relies on axisymmetric deformation models
Stretching methods		N/A	Submicron [202]	Controlled, large‐deformation loading; Simulate physiological mechanical stimuli	Require strong adhesion; Difficult to quantify the exact, absolute forces exerted directly on the nucleus; Indirect force application to the nucleus (mediated via focal adhesions and the cytoskeleton).
Passive imaging techniques	Particle tracking	N/A	Nanometer‐scale (∼10 nm) [203]	Completely passive and nondestructive; Capable of simultaneous, real‐time mapping of spatial mechanical heterogeneity.	Assume thermal equilibrium, which is often violated in active living cells; Require introduction of exogenous beads.
	Brillouin microscopy	N/A	Submicron [107]	Non‐destructive; Submicron spatial resolution; Label‐free, 3D nuclear stiffness mapping in living cells	Measures the high‐frequency longitudinal modulus, differing from the standard Young's modulus; Signals are highly sensitive to local hydration and refractive index variations.

### 3.1 Indentation and Compression Methods

#### 3.1.1 Atomic Force Microscopy

AFM quantifies nuclear mechanical properties by measuring the bending of a cantilever as it indents the nuclear surface (Figure 2a,b). In AFM‐based nanoindentation, a piezoelectrically controlled cantilever probe approaches and indents the nucleus, causing local deformation of the nuclear envelope. Both sharp and spherical probe geometries are commonly used, with sharp probes suited for high‐spatial‐resolution local measurements, whereas spherical probes are preferred for more averaged measurements of nuclear mechanics. Mechanical resistance from the nucleus leads to cantilever deflection, which is accurately monitored by detecting the angular displacement of a laser beam reflected off the cantilever onto a position‐sensitive photodiode. This setup achieves exceptional force sensitivity (tens of piconewtons to hundreds of nanonewtons [64]) and a spatial resolution of 0.5–1 nm [65]. This setup generates high‐fidelity force–indentation curves that, when fitted with contact mechanics models (e.g., Hertz or Sneddon), yield quantitative maps of local Young's moduli and viscoelastic parameters. This localized mode of interrogation is particularly valuable for resolving spatial variations in nuclear mechanics and mapping subnuclear mechanical heterogeneity [66]. However, it has intrinsically low throughput, and the anisotropic nuclear deformation under indentation leads to direction‐dependent mechanical readouts [67].

AFM has provided critical insights into nuclear mechanics, elucidating how nuclear stiffness responds to chromatin compaction [68], DNA damage [18], and variations in lamin A/C expression [69]. For instance, AFM enables high‐resolution spatial mapping of mechanical heterogeneity within the nucleus, revealing distinct mechanical contributions from the nuclear lamina and chromatin [70]. This capability has significantly advanced our understanding of nuclear softening mechanisms associated with mechanical perturbations and laminopathies—disorders linked to defects in nuclear lamina proteins [71]. Furthermore, AFM‐based nanoindentation studies of isolated nuclear envelopes reveal substantial stiffness heterogeneity, notably demonstrating that nuclear pore complexes exhibit markedly higher stiffness than surrounding membrane areas, underscoring their specialized structural roles [72].

#### 3.1.2 Compression Methods

Compression methods impose controlled, global deformation fields upon intact cells or isolated nuclei, serving as a robust platform for determining mechanical and viscoelastic properties (Figure 2a,c). Unlike AFM, which probes local surface stiffness, compression methods—ranging from single‐cell parallel plates to microfluidic confinement—quantify the whole‐organelle bulk modulus and the time constant [73]. By coupling compression measurements with finite‐element analysis, global force–deformation responses can be translated into intrinsic nuclear elastic properties, allowing for the quantitative estimation of parameters such as Young's modulus. For example, integrating microplate compression experiments on individual endothelial cells with finite‐element modeling has revealed that the nucleus is markedly stiffer than the surrounding cytoplasm, playing an important role in the global cellular resistance against compressive loading [74]. In contrast to AFM, compression methods are easy to implement, and their simple parallel‐plate geometry provides a uniform uniaxial stress field. However, their spatial and force resolution are limited, and plate‐based setups often require complex chemical surface treatments.

Critically, these methods have illuminated the context‐dependent mechanics of the nucleus. For instance, unconfined compression studies on single chondrocytes, combined with computational modeling, showed that when measured in situ with an intact cytoskeleton, the difference in elastic modulus between the nucleus and cytoplasm is significantly reduced compared to measurements on isolated nuclei. This result is associated with mechanical coupling between the cytoskeleton and the nucleus, as the intact cytoskeleton in whole cells alters the apparent mechanical properties of the nucleus measured under compression [75]. Furthermore, modern compression platforms have evolved into high‐throughput mechanotyping systems capable of screening thousands of cells simultaneously, making them indispensable for identifying mechanical biomarkers in cancer diagnostics and drug screening [76, 77].

### 3.2 Tweezer Methods

#### 3.2.1 Optical Tweezers

OT operate on the principle of photon momentum transfer, utilizing highly focused laser fields to generate steep electromagnetic gradients capable of trapping dielectric micro‐ and nanoparticles (Figure 2a,d). The experimental setup typically employs a high‐numerical‐aperture microscope objective to focus a near‐infrared laser, generating a 3D optical potential well. This noninvasive manipulation enables the precise application of localized forces (typically 0.1–100 pN [65]) with nanometer positioning accuracy and sub‐millisecond temporal resolution, making OT particularly well suited for probing high‐frequency mechanical responses. Another advantage of OT is its ability to perform high‐bandwidth, multidimensional manipulation of individual molecules or organelles without physical contact from a mechanical probe [78, 79]. However, the technique faces intrinsic limitations: the high photon flux at the trap focus can induce local heating and photothermal toxicity, potentially perturbing delicate biological systems, and the maximum achievable force is generally lower than that of AFM, limiting its utility for probing stiff macroscopic tissues.

OT have been deployed to probe mechanical properties across multiple length scales, from the global envelope to single chromatin fibers. By manipulating endocytosed dielectric beads positioned near the nuclear periphery, OT can function as an intracellular actuator to deform the nuclear envelope from within, extracting viscoelastic moduli while preserving the native cellular environment [71]. Due to their exceptional sensitivity, OT have significantly advanced the understanding of chromatin mechanics, revealing the force‐extension behaviors of nucleosomal and non‐nucleosomal chromatin fibers [89] and highlighting their role in nuclear mechanical properties [80]. Additionally, OT have shown that mechanical detachment of chromatin from the inner nuclear membrane enhances nuclear deformability by reducing nuclear stiffness [81].

#### 3.2.2 Magnetic Tweezers

MT operate by generating controlled magnetic field gradients—typically via permanent magnets or electromagnets—to manipulate superparamagnetic microbeads tethered to biological structures (Figure 2a,e). Unlike optical traps that rely on photon momentum, MT exert force based on the magnetic susceptibility of the probe, enabling a vast dynamic range from femtonewtons to approximately 1 nN [82]. The primary advantage of this modality is its intrinsic biocompatibility: magnetic fields penetrate biological tissue without attenuation or heating, thereby eliminating the phototoxicity and thermal noise associated with intense laser focus. This feature allows for “force‐clamp” spectroscopy, where constant loads can be applied stably over extended timescales (hours) to measure slow viscoelastic creep or rare equilibrium transitions. In addition, MT provide the distinct advantage of applying both linear tension and rotational torque, making them well‐suited for studying DNA supercoiling and chromatin fibers. Moreover, multiple beads can be tracked simultaneously, enabling higher‐throughput measurements than single‐target manipulation. However, standard MT setups rely on video‐based particle tracking, in which the camera frame rate constrains the measurement bandwidth (the highest frequency of motion that can be resolved) and image‐based localization accuracy constrains displacement sensitivity, thereby reducing the ability to directly resolve very fast or very small motions compared to the photodiode detection used in OT [65].

MT have been widely used to characterize chromatin fiber mechanics [83], detect nucleosome unwrapping transitions at low forces (∼3 pN) [84], and provide high‐resolution measurements of DNA elasticity at the nanometer scale [85, 86]. At the micrometer scale of the cell nucleus, MT have enabled direct probing of nuclear mechanical properties in intact cells, revealing spatial heterogeneity and anisotropy of apparent nuclear stiffness under piconewton‐scale loading. MT further enabled the quantification of nuclear viscosity, showing that while the effective elastic modulus increased under cyclic force application, the nuclear viscosity remained nearly unchanged [87].

### 3.3 Micropipette Aspiration

Micropipette aspiration serves as the established gold standard for characterizing the continuum mechanics of the cell nucleus. The technique employs a precision‐forged glass capillary (typically 1–3 µm inner diameter [88]) connected to a hydrostatic or pneumatic pressure controller (Figure 2a,f). By applying controlled negative pressure (suction), a specific portion of the nuclear surface is drawn into the pipette channel. The resulting deformation is monitored in real‐time via microscopy to generate time‐resolved creep compliance curves (deformation under constant stress) [89] or stress‐relaxation profiles [90]. By modeling the nucleus as a viscoelastic solid (e.g., Kelvin–Voigt [91] or Standard Linear Solid models [89]) or a liquid drop—and applying Laplace's law to account for surface tension—micropipette aspiration enables the extraction of fundamental material parameters, including the elastic modulus, viscosity, and cortical surface tension [92, 93]. Micropipette aspiration is well‐suited for probing nuclear envelope mechanics, particularly lamin‐dependent elasticity [94], but it remains a single‐cell method with low throughput and requires skilled operation [95].

Micropipette aspiration is compatible with fluorescence microscopy and has been widely employed to investigate nuclear mechanics, particularly in the context of laminopathies, chromatin remodeling, and lamin deficiencies [96, 97]. Micropipette aspiration experiments have revealed distinct nuclear deformation patterns, including deformation plateaus or continuous deformation under constant pressure, reflecting variability in chromatin organization, lamin composition, and nuclear viscoelastic behaviors [98]. Furthermore, recent advancements have expanded micropipette aspiration to directly characterize mechanical properties of subnuclear compartments, showing that nucleolar structures—such as the GC and the DFC—exhibit distinct viscoelastic responses; notably, the DFC demonstrates higher viscosity and slower relaxation dynamics compared to the GC [99]. These findings highlight the capability of micropipette aspiration to dissect the viscoelastic properties of the nuclear interior with high spatial resolution.

### 3.4 Stretching Methods

Stretching methodologies are designed to impose controlled tensile deformation fields on whole cells, serving as the primary tool for mimicking the macroscopic mechanical loads experienced by tissues such as muscle, lung, and skin. These assays generally employ two distinct configurations: substrate‐based stretching, where adherent cells are cultured on flexible elastomeric membranes (e.g., silicone) subjected to defined uniaxial or biaxial extension, and microstretching, which applies feedback‐controlled tensile loads directly to suspended spherical cells (Figure 2a,g). The former relies on indirect force transmission—stress propagates from the substrate through focal adhesions and the cytoskeleton to the nucleus—while the latter enables well‐defined deformation independent of substrate constraints. The major advantage of these platforms is their ability to simulate physiological strain regimes (large deformations) in a controlled manner. However, they are inherently limited: substrate methods require robust cell adhesion for efficient force transfer, and the exact, absolute forces exerted directly on the nucleus remain difficult to quantify.

When integrated with computational modeling, stretching techniques provide quantitative relationships between externally applied tensile strain and spatially resolved, time‐dependent nuclear mechanics. For instance, finite element analyses of cells subjected to substrate stretching reveal significant spatial heterogeneity in intranuclear strain, demonstrating that distinct nuclear regions deform differently under identical external loading [100]. Additionally, microstretching experiments on suspended endothelial cells have provided complementary insights, with computational modeling and creep–recovery analysis indicating nuclear viscoelastic behaviors consistent with a Kelvin–Voigt solid model [101]. Together, these stretching methods substantially deepen the understanding of nuclear mechanical responses under physiologically relevant mechanical stimuli.

### 3.5 Passive Imaging Techniques

#### 3.5.1 Particle Tracking

Using time‐lapse fluorescence microscopy, the trajectories of extrinsic tracers (e.g., quantum dots, viral particles) or intrinsic organelles are recorded with nanometer precision. The analysis can either calculate the time‐averaged mean squared displacement to characterize the mode of transport, or simultaneously monitoring high‐density ensembles of tracer particles dispersed throughout the nuclear interior (Figure 2a,h). By applying the Generalized Stokes–Einstein Relation, these kinematic data are transformed into frequency‐dependent viscoelastic moduli (*G'* and *G''*). The primary advantage of particle tracking is its ability to map local mechanical heterogeneity at the nanoscale without exerting external stress, thereby preserving the cell's physiological state. However, particle tracking typically assumes thermal equilibrium, which is often violated in active living cells, and many implementations require the introduction of exogenous beads, which may perturb the native nuclear environment.

Particle tracking provides a quantitative framework to probe nuclear mechanics under both physiological and disease‐associated conditions. At the nanoscale, a recent study used particle tracking to characterize dynamic shifts in nuclear viscoelasticity during mesenchymal stem cell differentiation, demonstrating correlations between transient nuclear stiffening and softening events and chromatin reorganization processes [102]. At the microscale, particle tracking has been applied to connect chromatin dynamics to DNA damage and repair, DNA replication and transcription in live cell nuclei [103]. Furthermore, particle tracking exploits membraneless organelles—such as PML nuclear bodies and Cajal bodies—as endogenous rheometers. The diffusive behavior of these PML nuclear bodies acts as a sensor for the steric hindrance and accessibility of the surrounding interchromatin channels, providing a readout of how the physical crowding of the nucleoplasm regulates the targeting of functional complexes to specific nuclear subcompartments [104]. Beyond these mechanistic studies, particle tracking has proven highly effective for disease‐specific mechanical phenotyping. In hematological malignancies, particle tracking analyses have revealed that nuclei from high‐risk leukemia cells exhibit significantly increased nuclear viscosity compared to those of normal lymphocytes [105]. This capability to rapidly map viscoelastic changes across entire cell populations positions particle tracking as a promising tool for developing high‐throughput mechanodiagnostic assays.

#### 3.5.2 Brillouin Microscopy

Brillouin microscopy is an advanced imaging technique that enables noncontact elastography of the cell nucleus. The technique relies on the inelastic scattering of incident laser light by thermally induced acoustic phonons (hypersonic sound waves) propagating within the sample. The interaction results in a spectral frequency shift—the Brillouin shift—which is directly proportional to the local acoustic velocity. This metric provides a quantitative readout of the material's longitudinal elastic modulus and density, offering a distinct advantage over contact‐based methods that measure the quasistatic Young's modulus. By scanning the sample in 3D, Brillouin microscopy generates high‐resolution stiffness maps with submicron spatial precision (Figure 2a,i) [106, 107]. A key advantage of this modality is its label‐free nature, allowing for the interrogation of native nuclear mechanics in living cells without the need for exogenous tracers or physical perturbation. However, the longitudinal modulus (measured at GHz frequencies) is sensitive to hydration [108], necessitating careful calibration for comparative analysis.

Recent studies employing Brillouin microscopy have effectively distinguished the longitudinal elastic modulus of nuclei from adjacent cytoplasmic regions [109]. Furthermore, combining Brillouin microscopy with fluorescence imaging techniques enables correlative analyses, revealing relationships among chromatin organization, lamina architecture and the longitudinal modulus of the nucleus [110, 111, 112]. Notably, integrated studies using this approach have characterized the mass density and mechanical properties of nucleoli, identifying significantly higher Brillouin shifts indicative of their less fluid‐like, mechanically stiffer nature compared to surrounding nucleoplasmic regions [113, 114].

Together, these experimental techniques constitute a comprehensive toolkit for investigating nuclear mechanics across multiple scales, with each method offering distinct strengths: indentation and compression methods deliver localized normal loads to quantify nuclear stiffness, elasticity and viscoelastic responses; tweezer methods apply calibrated forces at subcellular resolution to probe chromatin fiber mechanics, nucleosome transitions and DNA elasticity; micropipette aspiration characterizes nuclear deformability, yield stress and viscoelastic recovery by aspirating nuclei under controlled negative pressure; stretching methods assess nuclear deformation and mechanical heterogeneity under controlled uniaxial strain supported by finite‐element analyses and creep–recovery modeling; and passive imaging techniques provide noninvasive access to nuclear material properties by quantifying tracer‐particle fluctuations or Brillouin frequency shifts to yield viscoelastic metrics, stiffness maps, and condensate mechanics across multiple spatial scales.

## 4 Modelling of Nuclear Mechanics

The mechanical properties of the cell nucleus profoundly influence cellular functions, from gene expression to cell behavior. Rather than being uniform, these properties emerge from intricate interactions among diverse nuclear components across multiple length scales, including chromatin organization, lamin network architecture, and active molecular remodeling processes [115, 116, 117]. Mechanical models bridge microscopic mechanisms with observable macroscopic properties, such as stiffness, stress relaxation, poroelastic responses, and envelope rupture [118]. Such models transform qualitative observations into quantitative, testable predictions, aiding in the interpretation of experimental data and forecasting how mechanical alterations impact nuclear transport and gene regulation [119, 120]. These frameworks further facilitate the translation of nuclear mechanics into clinically relevant biomarkers and inform the development of targeted mechanical or molecular interventions [121, 122]. Nuclear mechanical models can be classified based on the scales they target, ranging from molecular‐level determinants to intermediate‐scale polymer networks, ultimately culminating in integrated models that capture the emergent mechanical behavior of the entire nucleus.

### 4.1 Mechanics of DNA

DNA mechanics underpins essential nuclear behaviors, from gene regulation to overall nuclear integrity. Understanding DNA mechanics has prompted the development of theoretical models spanning multiple length scales, each tailored to specific biophysical contexts.

**FIGURE 3:**
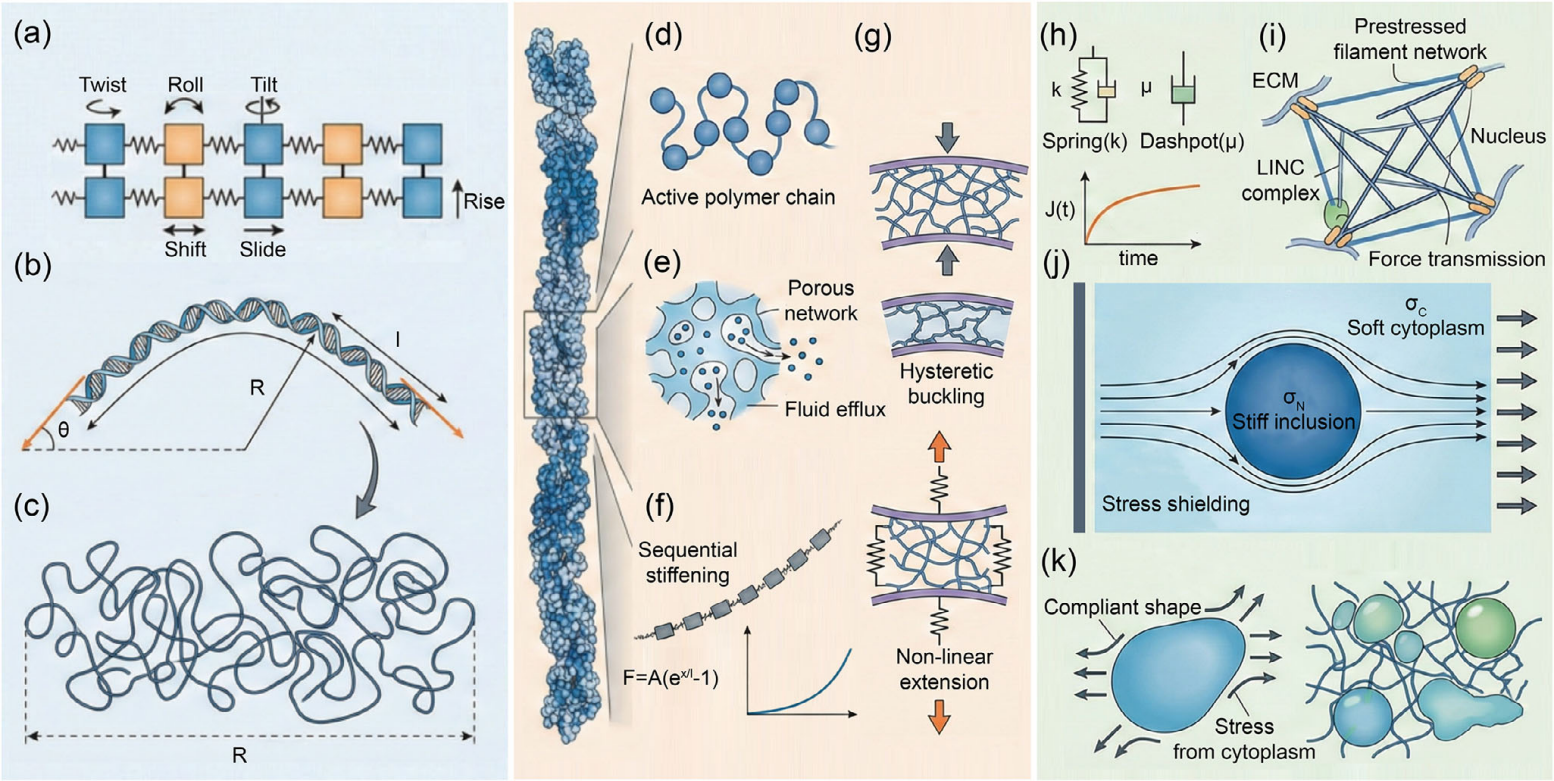
Overview of multiscale mechanical models of the cell nucleus. (a) The rigid base‐pair model represents DNA as rigidly connected segments, describing detailed interactions at the smallest scales. (b) The worm‐like chain model describes DNA as a semiflexible polymer characterized by its bending rigidity and persistence length. (c) The flexible polymer model depicts DNA behavior dominated by entropic elasticity and thermal fluctuations. (d–f) Complex chromatin mechanics are described by models such as coarse‐grained active polymer frameworks, poroelastic models characterizing fluid movement through a soft porous network, and the Hierarchical Chain Model which explains sequential stiffening arising from structural heterogeneity. (g) The nuclear lamina is modeled as a cross‐linked biopolymeric shell, capturing nonlinear mechanical behaviors including stiffness and hysteretic buckling. (h) Viscoelastic frameworks capture the nucleus's dual ability to store (elastic) and dissipate (viscous) mechanical energy. (i) The tensegrity model integrates the nucleus into the cellular cytoskeletal network for force transmission. (j) Computational approaches like the elastic phase field method are used to study phenomena such as stress shielding. (k) Alternative models suggest nuclear shape is dictated by viscous stresses from the cytoplasm (viscous coupling) or explore the mechanical interplay between the elastic chromatin network and liquid‐like nuclear condensates.

At the smallest scales, the rigid base‐pair model represents DNA as a sequence of rigidly connected segments, effectively describing detailed sequence‐dependent interactions with proteins (Figure 3a). The rigid base‐pair framework expresses the deformation energy *E* of adjacent base pairs through a harmonic potential [123, 124]:

$$E=E_{0}+\frac{1}{2}\sum_{i=1}^{6}\sum_{i=1}^{6}f_{ij}\Delta u_{i}\Delta u_{j}$$

where *f_ij_* denotes elastic constants of the matrix, Δ*u_i_* and Δ*u_j_* represent the instantaneous fluctuations of the six geometric degrees of freedom of a base‐pair step from its equilibrium state. These fluctuations cover the three rotational parameters (Twist, Roll, Tilt) and the three translational parameters (Shift, Slide, Rise). On a relatively large scale, the worm‐like chain model is a semiflexible polymer model that describes DNA on the scale of tens to hundreds of nanometers (50–100 nm) (Figure 3b), where bending and twisting elastic degrees of freedom become apparent [125, 126]:

$$E\;\left(\theta \right)=\frac{\kappa L}{2R^{2}}=\frac{\kappa \theta^{2}}{2L}$$

where *E*(θ) is the energy required to bend a DNA of length L through an angle θ and a radius of curvature *R*/*L*, and κ is the bending rigidity. A larger scale on top of this classic model is the flexible polymer model (Figure 3c). The DNA exhibits an entropic elasticity and the polymer's behavior is dominated by random thermal fluctuations, leading to a coiled, random configuration in the absence of external forces [127]. The ideal chain limit in this model relates the chain's overall dimension to its bending rigidity in a force‐free state:

$$\langle R^{2}\rangle \approx 2Ll_{b}$$

where *R* ^2^ is the mean squared end‐to‐end distance of the polymer, a statistical measure of the overall size of the coiled chain. *L* is the contour length of the polymer and *l_b_* is the bending persistence length. Understanding the relationships between these independent models at different scales is also crucial. One recent framework coarse‐grains the rigid base‐pair model into a generalized worm‐like chain and then into a flexible polymer limit [128]. At each step short‐range degrees of freedom are integrated out to yield effective bending, torsional, and entropic stiffnesses. The work shows that elasticity is scale‐dependent and that rotational–translational couplings set the long‐wavelength persistence lengths.

### 4.2 Modeling the Chromatin and Nuclear Lamina

While those foundational models describe naked DNA, they are insufficient to explain the behavior of chromatin fibers within the nucleus. More complex models are therefore required. An approach seeks to explain bulk properties through specific molecular interactions, such as modeling Heterochromatin Protein 1α as a physical cross‐linker that stiffens the chromatin mesh into a polymer gel [129]. It is also crucial to recognize the limitations of certain models. For instance, classic rheological frameworks, such as the Rouse model, have been shown to be insufficient for recapitulating the observed dynamics of chromatin movement [130], revealing that its mechanical behavior is dominated by a structural hierarchy and protein interactions not captured in simple polymer frameworks.

Chromatin acts not only as a genetic repository but also as a significant structural and mechanical component, displaying behaviors characteristic of both fluids and solids depending on context and timescale. At molecular and mesoscale levels, chromatin can be effectively modeled using coarse‐grained polymer frameworks. For instance, the active Zimm chain model represents chromosomes as active polymer chains interacting hydrodynamically (Figure 3d), providing insights into how euchromatin activity influences heterochromatin compaction and spatial organization [131]. On the continuum scale, the poroelastic model, for instance, is relevant for processes that involve fluid–solid separation and true volume changes (Figure 3e). This model treats chromatin as a soft porous material, characterizing the dynamics of fluid (nucleoplasm) movement through the chromatin network, such as in response to osmotic shocks or during slow, long‐term consolidation. It is defined by [132, 133]:

$$\tau =\frac{r^{2}}{D}$$

where τ is the characteristic timescale for the fluid efflux in response to compression, *D* is the poroelastic diffusion coefficient reflecting chromatin permeability, and *r* is the radius of the nucleus. More sophisticated models, such as the compressible polymer gel model, further incorporate the mechanical and chemical properties of chromatin subcompartments. By linking chromatin condensation and mechanical deformation, these advanced frameworks can predict unique mechanical responses, such as auxetic behavior characterized by negative Poisson's ratios [134, 135]. This diversity of theoretical approaches underscores ongoing debate about the precise material state of chromatin, which has been variously described as a liquid [132], a simple viscous medium [130], or a solid [129] on a similar scale.

Beyond these simple homogeneous descriptions, the Hierarchical Chain Model has emerged to explain chromatin's complex mechanics by focusing on its intrinsic structural heterogeneity (Figure 3f). This model posits a “sequential stiffening” mechanism, where low forces deform the softest components, and stiffer elements are progressively recruited at higher forces. Depending on the system, this hierarchical principle manifests in distinct mathematical forms. For interphase nuclei, it produces an exponential force‐extension curve [136, 137]:

$$F=A\left(e^{x/l}-1\right)$$

where *F* is the force, *x* is the extension, and *A* and *l* are scaling parameters reflecting the stiffness range of the hierarchical elements. In mitotic chromosomes, the same principle explains the “anomalous” weak stiffening, where the differential stiffness *K* scales with force *F* as *K*∝*F* ^γ^, with a characteristic scaling exponent γ < 1. This hierarchical framework successfully captures the mechanical softening observed after MNase digestion, directly linking chromatin's heterogeneous structure to its function [137].

At the subcellular scale, the other type of model addresses how molecular assemblies define the mechanical properties of nuclear substructures, particularly the nuclear lamina. Often represented as a thin elastic or viscoelastic shell, the lamina is frequently modeled using continuum‐based or biopolymeric approaches. The biopolymeric shell model, specifically, treats the lamina as a cross‐linked polymer mesh, effectively capturing essential mechanical behaviors including stiffness, elasticity, and deformation dynamics (Figure 3g). This approach accurately predicts characteristic mechanical responses such as the hysteretic buckling transitions that occur under mechanical stretching or compression, providing critical insight into lamina‐associated morphological changes and mechanical failure modes observed experimentally [129, 138]. In this model, the mechanical response under tension is described by:

$$\;\frac{\Delta L\left(F\right)}{L}=\left(\frac{1/k_{1}+F/k_{2}F_{1}^{*}}{1+F/F_{1}^{*}}\right)\;\frac{F}{L}$$

where Δ*L*(*F*) is the extension of the lamina under applied tension 𝐹, *L* is the initial length corresponding to the mean lamina diameter at zero tension, *k*_1_ and *k*_2_ are spring constants describing linear elastic regimes at small and large tensions respectively, and $F_{1}^{*}$ is the characteristic force defining the transition between these regimes. At a more detailed scale, filament‐level models of lamin networks further elucidate how specific mesh topologies and intrinsic lamin filament properties collectively yield the observed nonlinear, emergent mechanical behaviors of the nuclear lamina structure [71, 139].

### 4.3 Emergent Mechanics of the Whole Nucleus

While understanding individual components is crucial, predicting the nucleus's role in cellular processes, such as migration, differentiation, and disease, requires scaling up to the organelle level. At this scale, models condense the complex internal architecture into a set of effective material properties, allowing for the analysis of nuclear deformation, stress distribution, and mechanotransduction in response to physiological forces.

A primary emergent property of the nucleus is its viscoelasticity—the ability to both store and dissipate mechanical energy (Figure 3h), exhibiting characteristics of an elastic solid and a viscous fluid [140]:

$$J\left(t\right)=\frac{1}{k_{M}}+\frac{1}{k_{KV}}\left(1-e^{-t/\tau }\right)+\frac{t}{\mu_{M}}$$

where *J*(*t*) is creep compliance, response time is τ  = µ_*KV*_/*k_KV_* . *k* and µ correspond to the elastic coefficient and the viscosity coefficient, and *M* and *KV* represent the Maxwell module and the Kelvin–Voigt module. By systematically perturbing nuclear components, they defined the distinct contributions of A‐type lamins, B‐type lamins, and chromatin to the overall viscoelastic response, providing a concise framework for predicting how molecular alterations map to changes in organelle‐level mechanics. Building on this, a standard linear solid model is employed to capture the nonmonotonic mechanical response of the nucleus in vivo [141] (Figure 3i):

$$\varepsilon =-\nu \frac{\sigma_{c}}{k_{c}}+\frac{\sigma_{t}}{k_{t}}\left(1-e^{-t/\tau }\right)$$

the first term represents the instantaneous elastic response, and the second term represents the time‐dependent viscoelastic response. This model successfully characterizes the instantaneous elastic deformation, subsequent creep, and eventual recovery observed in experiments.

The nucleus is a central hub in the cell's mechanotransduction network. The tensegrity model provides a foundational concept, positing a continuous network of prestressed filaments that transmit forces from the extracellular matrix (ECM), through focal adhesions and the cytoskeleton, directly to the nuclear lamina and chromatin [134, 142]. This integration gives rise to powerful chemomechanical feedback loops. For example, increased ECM tension enhances actomyosin contractility, which in turn deforms the nucleus, alters chromatin organization, and modulates the shuttling of epigenetic factors. These nuclear changes can then drive altered gene expression that remodels the ECM, completing the feedback cycle [143, 144]. Computational approaches, such as the elastic phase field method (Figure 3j), simplify this intricate system by modeling the nucleus as a stiff elastic inclusion within a softer cytoplasm [145]:

$$\Sigma \left(\rho ,\psi \right)=\left[h\left(\rho \right)-h\left(\psi \right)\right]\sigma^{C}+h\left(\psi \right)\sigma^{N}$$

where ***σ*** ^***C***^ and ***σ*** ^***N***^ are stress tensor in cytoplasm and nucleus, ρ and ψ are order parameters, and *h*(*x*) is the weight function. This is particularly useful for studying phenomena like stress shielding, where the nucleus is protected from external deformations. Offering an alternative perspective, the viscous coupling model challenges the view of a statically shaped nucleus [142] (Figure 3k) It hypothesizes that the nucleus is highly compliant due to an excess surface area of the nuclear lamina and that its shape is primarily dictated by viscous stresses transmitted from moving cell boundaries through the cytoplasm, particularly during dynamic processes like cell migration.

Finally, at the intersection of soft matter physics and nuclear biology, theoretical models are beginning to explore the interplay between chromatin mechanics and the liquid‐like phase separation of nuclear condensates. It is proposed that the elastic chromatin network can mechanically frustrate or constrain the growth and coalescence of these membraneless organelles [146]. This concept introduces a fascinating link between the material state of chromatin and the thermodynamic regulation of key nuclear processes, suggesting that the nucleus's mechanical properties not only govern its response to external forces but also actively shape its internal biochemical landscape.

## 5 Translational Implications of Multiscale Nuclear Mechanics

**FIGURE 4:**
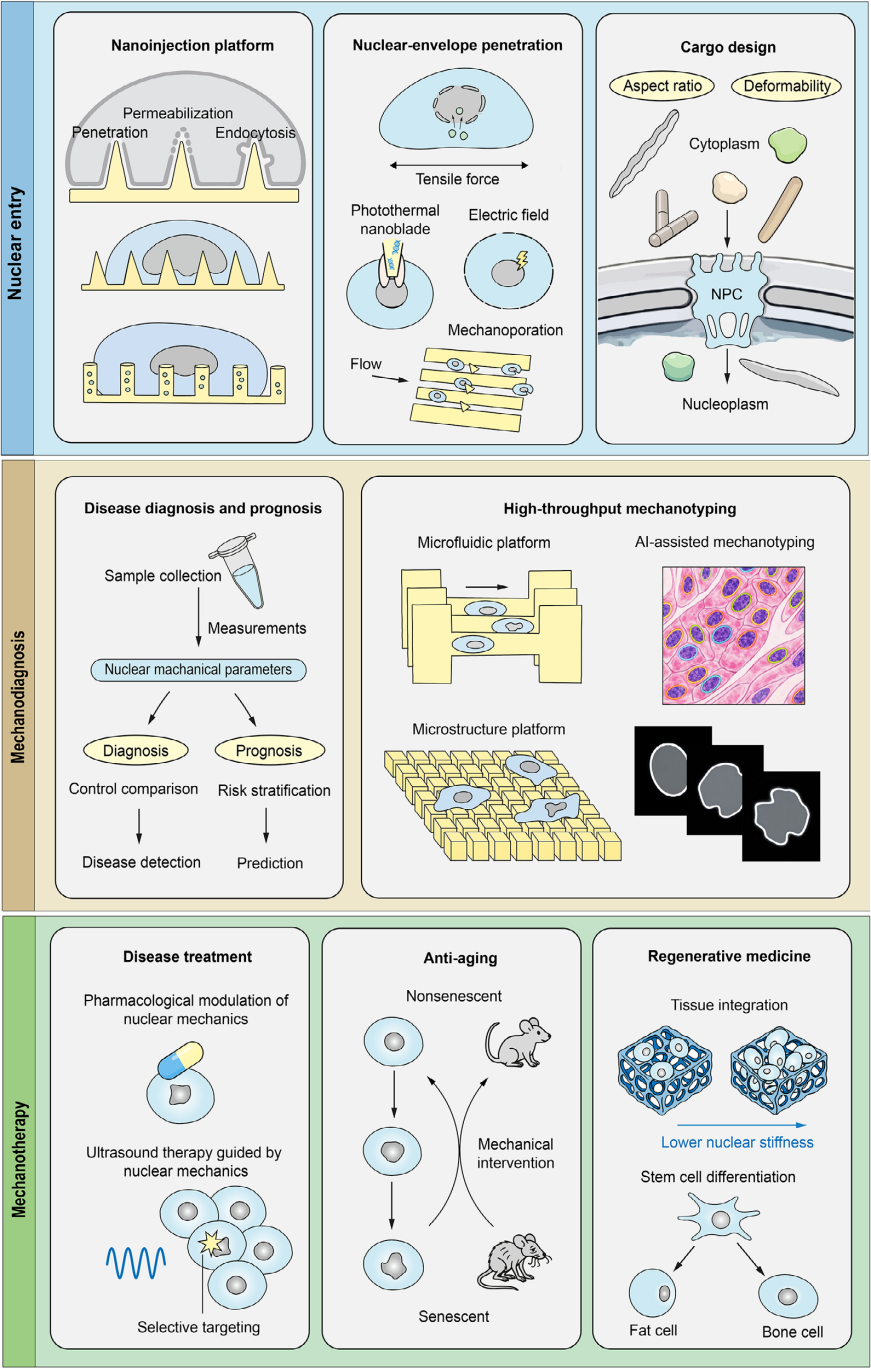
Biomedical applications of multiscale nuclear mechanics. Insights into multiscale nuclear mechanics have enabled three major applications: nuclear entry, mechanodiagnosis, and mechanotherapy. In nuclear entry, the mechanical properties of the nuclear envelope guide the design of delivery strategies, including nanoinjection, nuclear‐envelope penetration, and cargo design based on geometric and mechanical properties. In mechanodiagnosis, high‐throughput and AI‐assisted nuclear mechanotyping supports disease diagnosis and prognosis. In mechanotherapy, nuclear mechanics is modulated or exploited for disease treatment, anti‐aging and regenerative medicine.

The elucidation of multiscale nuclear mechanics is now transitioning from fundamental biophysics to a cornerstone of translational medicine, offering novel paradigms for nuclear entry, mechanodiagnosis and mechanotherapy (Figure 4). In nuclear entry, the mechanics of the nuclear envelope as a transport barrier inform delivery design. In mechanodiagnosis, nuclear mechanical phenotypes provide quantitative, label‐free readouts of pathological states. In mechanotherapy, nuclear mechanics can be modulated or exploited for therapeutic benefit. Collectively, these convergence technologies promise to transform our understanding of nuclear physics into actionable clinical strategies for disease treatment, anti‐aging intervention and regenerative medicine.

### 5.1 Mechanics‐Inspired Strategies for Nuclear Entry

#### 5.1.1 Nanoinjection Platform

Accurate delivery of therapeutic agents is critical for effective intervention in complex pathological conditions. In this context, nuclear‐targeted delivery has emerged as a promising strategy, especially in gene therapy and cancer treatment. The nuclear mechanics has important translational implications for nanomedicine and bioengineering, as the efficacy of many nuclear entry strategies depends on whether cargoes can overcome the mechanical barrier of the nuclear envelope [147]. To address this challenge, nanoinjection platforms are physical intracellular delivery technologies that use vertically configured high‐aspect‐ratio nanostructures to penetrate the plasma membrane with minimal perturbation and toxicity [148, 149]. These platforms include nanoneedles, and their electroactive analogs, nanotubes, and vertical nanoprobes. Nanoneedles are arrays of vertical high‐aspect‐ratio nanostructures that mediate delivery through three main interfacing mechanisms: mechanical penetration, permeabilization, and endocytosis [149, 150]. When coupled with electrical stimulation, these electroactive nanostructures can induce transient ‘holes’ in the cell membrane, providing faster and more direct intracellular access [151, 152]. However, current nanostructure‐mediated electroporation platforms enable only cytosolic delivery and cannot achieve nucleus‐targeted delivery [153]. Unlike nanoneedles, nanotubes are hollow nanostructures designed for sustained release; by exploiting their inner cavity as a reservoir, they can co‐load multiple bioactive cargoes without surface functionalization and support nanoelectroporation‐ or optoporation‐mediated delivery [154, 155, 156]. In addition, vertical nanoprobes can be used not only for intracellular delivery but also for transmembrane sampling, intracellular sensing, and electrical recording [153, 154]. These platforms collectively provide a basis for nucleus‐targeted delivery across the nuclear‐envelope barrier.

Nanoinjection platforms can establish interfaces that reach the depth of the nucleus with appropriate engineering [157]. When cells interface with nanoneedle or nanoprobe arrays, the nucleus can undergo marked deformation, with nuclei becoming more rounded, less flattened, and in some cases showing nuclear membrane remodeling or even possible penetration of the nuclear envelope [158, 159]. Nanoprobe‐induced deformation is strongly geometry dependent, as nanoprobes longer than 1.4 µm can deform the nuclear envelope, and decreasing the radius, increasing the height, or increasing the separation of nanoprobes induce greater nuclear deformation, while tip diameter substantially affects the depth and shape of deformed nuclear membranes [154]. Meanwhile, studies on silicon nanoneedle arrays have shown dramatically increased membrane curvature, which may impose sufficient force on the nucleus to cause rupture [158]. These perturbations are also accompanied by changes in lamin localization, with lamin A accumulating at nanoprobe tips, whereas lamin B is less sensitive to the stimulus and remains uniformly distributed. Moreover, the stiffness of the probe also influences intracellular access, as stiffer materials are more likely to breach the membrane under applied force, leading to a more effective interface [154]. Therefore, elucidating the mechanics that govern nuclear entry, particularly the mechanical properties of the nuclear envelope, will guide the rational design of nucleus‐targeted delivery systems that more effectively overcome the nuclear‐envelope barrier.

#### 5.1.2 Mechanical Membrane and Nuclear‐Envelope Penetration

Beyond the nanoinjection approaches, mechanical strategies for nuclear entry also include force dependent regulation of transport through nuclear pore complexes within the nuclear envelope, as well as transient physical opening of the nuclear envelope coupled with photothermal nanoblade, electric field, or microfluidic constriction. When substrate stiffness increases, tensile forces are transmitted to the nucleus through integrin‐based adhesions and the LINC complex, which flatten the nucleus, enlarge the apparent size of nuclear pore complexes, and promote the nuclear import of Yes‐associated protein and other cargoes [10, 160]. Conversely, reduced nuclear‐envelope tension during energy depletion or hyperosmotic shock drives nuclear pore complex constriction and is accompanied by reduced nucleocytoplasmic diffusion [13]. Transient physical opening of the nuclear envelope has been achieved by photothermal nanoblade delivery, disruption and field enhanced delivery, and nuclear envelope mechanoporation. In photothermal nanoblade delivery, a micropipette is positioned above the nucleus, with a pulsed laser generating a localized cavitation bubble that opens the plasma membrane and nuclear envelope at the contact site, after which pressure‐driven flow transfers plasmid DNA directly into the nucleus [161]. In the disruption and field enhanced delivery platform, cells are first forced through microfluidic constrictions smaller than the cell diameter to mechanically disrupt the plasma membrane, and a downstream electric field then further disrupts the nuclear envelope and electrophoretically drives plasmid DNA into the nucleus [162]. In nuclear envelope mechanoporation, suspended cells are individually driven through microfluidic constriction channels, where the sharp‐tipped nanolancets that protrude from the bottom surface of the microchannel porate both the plasma membrane and nuclear envelope and allow exogenous plasmid DNA to immediately gain access to the nucleoplasmic space [163]. Collectively, these advances establish nuclear mechanics as a translational framework for delivery design, in which force mediated regulation of nuclear pore complexes and transient physical opening of the nuclear envelope can be converted into practical strategies for controlled nuclear access.

#### 5.1.3 Cargo Design for Deformation‐Based Transport

In addition to these membrane‐penetration strategies, effective nuclear delivery of therapeutic agents frequently employs nanocarriers engineered to navigate through NPCs. As the primary gatekeeper of nucleocytoplasmic exchange, the NPC presents a mechanically defined transport barrier whose properties directly inform nanocarrier design. AFM‐based nanomechanical mapping of intact NPCs reveals higher stiffness at the pore center, and shows that binding of nuclear transport receptors homogenizes the stiffness profile across the pore, supporting a gating mechanism based on modulation of inter‐Nup interactions [72]. Within this framework, intrinsic carrier properties (e.g., shape and mechanical deformability) determine how cargo physically interacts with and traverses this barrier.

These biophysical constraints have motivated the development of polymer‐ and peptide‐based nuclear‐targeting carriers with architectures optimized for passage through the nuclear pore. A recent study shows that high–aspect–ratio assemblies, such as rod‐like and worm‐like structures, can effectively translocate through nuclear pores and enhance the concentration of genetic and therapeutic agents within the nucleus [25]. Additionally, the mechanical stiffness of the carrier acts as a critical determinant, as soft particles can structurally deform and “squeeze” through the channel of the NPC. For example, deformable hollow organosilica nanocapsules undergo a spherical‐to‐oval morphology change during entry into human breast cancer MCF‐7 cells, enabling nuclear translocation and resulting in a 26‐fold increase in cellular uptake compared with undeformable organosilica nanospheres. Drug loading with doxorubicin further results in pronounced cytotoxicity, demonstrating the biomedical relevance of this approach [24]. Together, these findings establish a design framework in which nanocarrier geometry and deformability are tuned to the mechanical properties of the NPC, advancing the rational development of nucleus‐targeted therapeutics.

### 5.2 Mechanodiagnosis: The Nucleus as a Quantitative Label‐Free Marker

#### 5.2.1 Mechanical Biomarkers in Disease

Alterations in nuclear mechanics are closely linked to the onset of various diseases, including laminopathies such as Hutchinson–Gilford progeria syndrome (HGPS), lamin A/C cardiomyopathy, autosomal dominant leukodystrophy, and cancers. For instance, in HGPS, micropipette aspiration reveals significantly reduced creep compliance in patient‐derived nuclei, indicating a loss of viscous damping dominated by a rigid, defective lamina [164]. Similarly, AFM nanoprofiling of cardiomyocytes harboring the lamin A/C D192G mutation (associated with cardiomyopathy) demonstrates a measurable increase in Young's modulus compared to wild‐type controls [165]. Furthermore, autosomal dominant leukodystrophy, driven by lamin B1 overexpression, is characterized by nuclear hyper‐rigidity, offering a quantifiable biomarker for diagnosis [166]. Large‐scale nanoindentation studies consistently reveal that cancer cell nuclei are significantly more compliant, showing an approximate 60.3% reduction in stiffness, compared to normal cells [167]. This enhanced deformability facilitates invasion and affects chromatin remodeling [168]. These findings collectively suggest that nuclear mechanical parameters constitute quantitative descriptors of disease states across diverse pathological contexts.

Moreover, nuclear mechanics is closely linked to disease progression. Emerging evidence indicates that altered nuclear mechanical and structural states are associated with more aggressive disease behavior and adverse clinical outcomes. For example, in hematological malignancies, nuclei from high‐risk, relapsed acute lymphoblastic leukemia patients exhibit elevated viscosity and elasticity with high spatial heterogeneity, suggesting that nuclear viscoelasticity can serve as a nuanced prognostic indicator beyond simple stiffness [105]. Nuclear mechanical remodeling in confined 3D microenvironments can influence malignant cell behavior during disease progression [12, 169]. Jurkat cells selected after 3D growth conditions showed increased nuclear deformability and a more homogeneous stiffness across the nucleus, with reduced spatial heterogeneity in nuclear stiffness and increased mean stiffness, and also presented changes in apoptosis, sensitivity to chemotherapeutic drugs, and in vivo dissemination [12]. In addition, AI‐based segmentation and classification of nuclei from histological images have identified cell‐type‐specific nuclear features associated with genomic instability; for instance, larger fibroblast nuclei in breast cancer correlate with reduced progression‐free and overall survival [170]. Across multiple cancer types, automated chromatin texture analysis (nucleotyping) stratifies patient survival risk and retains prognostic significance even after multivariable adjustment in independent validation cohorts [171]. Collectively, these findings demonstrate that nuclear mechanical and morphological parameters extend beyond static disease descriptors, serving as dynamic indicators of disease progression and patient prognosis across diverse malignancies.

#### 5.2.2 High‐Throughput and AI‐Assisted Nuclear Mechanotyping

Nuclear mechanotyping is emerging as a label‐free strategy for high‐throughput phenotyping of disease states based on nuclear mechanical properties and deformability. Current approaches can be broadly divided into two categories: microfluidic platforms and microstructured platforms. Using a high‐throughput microfluidic micropipette aspiration platform, analysis of intact cells showed that fibroblasts from a patient with LMNA‐related dilated cardiomyopathy had more deformable and less viscous nuclei than matched control fibroblasts [172]. Microstructured platforms diagnose disease‐associated nuclear abnormalities by imposing defined topographic constraints and reading out the resulting nuclear shape changes with automated image analysis. A micropillar platform showed that cancer cells and noncancerous cells from the same tissue type could be distinguished by differences in nuclear deformability [173]. Likewise, microgroove substrates revealed abnormal nuclear deformation patterns in myoblasts from patients with LMNA mutations [174]. Collectively, these approaches translate nuclear mechanics and mechanically‐induced nuclear deformation into quantifiable readouts, thereby providing a basis for disease phenotyping and for the development of mechanics‐informed diagnostic platforms. Realizing this potential will require the establishment of standardized measurement protocols, robust data normalization strategies, and clear links between mechanical phenotypes and clinically meaningful outcomes. Moreover, integration with existing molecular and histopathological information will be essential to place nuclear mechanical parameters within broader diagnostic decision‐making frameworks.

In recent years, the convergence of AI with biophysics has given rise to “mechanomics”, a computational paradigm where deep learning models infer quantitative mechanical phenotypes directly from standard optical or histological images [175]. This approach enables high‐throughput, label‐free mechanotyping, thereby bypassing the limitations of low‐throughput direct force measurement. Recent advances in AI are transforming nuclear structural analyses into clinically actionable insights, particularly in the context of cancer diagnosis. For example, deep learning methods analyzing whole‐slide histopathological images have effectively differentiated normal and malignant tissues by extracting nuclear morphological features such as shape, size, color, and texture [170]. Specifically, AI‐driven detection of extreme nuclear lamina wrinkling has emerged as a cancer‐associated morphological marker, suggesting potential applications for automated cancer detection across multiple tissue types [122]. AI‐driven mechanotyping approaches currently focus on cellular‐scale mechanical parameters, such as cell stiffness inferred from optical images and benchmarked against AFM‐based stiffness measurements [175]. Integrating quantitative mechanical parameters such as nuclear stiffness and viscoelastic properties into these AI‐driven analyses could further anchor morphological observations to nuclear mechanics, improving the accuracy and robustness of early disease detection.

### 5.3 Mechanotherapy: The Nucleus as a Target for Treatment

#### 5.3.1 Perturbing Nuclear Mechanics for Disease Treatment

Therapeutic strategies leveraging nuclear mechanics encompass pharmacological interventions and mechanotherapy. For instance, farnesyltransferase inhibitors can partially reverse the increased nuclear stiffness observed in cells from patients with HGPS, underscoring the therapeutic potential of modulating nuclear mechanics [20]. Similarly, cisplatin treatment in cancer induces DNA damage and subsequent chromatin decondensation, resulting in decreased nuclear stiffness, increased nuclear envelope relaxation, and enhanced molecular diffusion [18]. These mechanical responses may inform new strategies for evaluating the effectiveness of cancer therapy and predicting drug resistance. Ultrasound therapy utilizes inherent differences in nuclear mechanics between cancerous and healthy cells to selectively target tumor cells with controlled physical stimuli. Using low‐intensity therapeutic ultrasound, AFM‐based morphology and stiffness maps were used to obtain quantitative estimations of the elastic stiffness of the cell nucleus and nuclear envelope and to inform a cell‐specific and experimentally informed dynamic model for frequency selection; guided by this modelling framework, ultrasound at 1 MHz was applied to cells in vitro and was found to amplify oscillations in cancer cells, with vibration‐induced stresses transmitted through the LINC complex to the nuclear lamina, leading to nuclear envelope rupture and accumulation of DNA damage [21].

#### 5.3.2 Regulating Nuclear Mechanics for Anti‐Aging

Beyond oncology, nuclear mechanics is emerging as a reliable biophysical target for anti‐aging interventions at cellular and tissue levels. At the cellular level, cyclic mechanical stretching has been shown to increase nuclear stiffness, induce nuclear envelope deformation, and promote DNA damage, collectively accelerating cellular senescence [22]. Conversely, interventions that soften the nucleus can mitigate nuclear deformation and DNA damage, delaying mechanically‐induced senescence [22]. At the tissue level, cardiomyocyte nuclei from aged Drosophila exhibit increased stiffness coupled with reduced expression of lamin C and lamin B. Experimental depletion of lamin C in young Drosophila cardiomyocytes replicates aging phenotypes, including nuclear stiffening, sarcomere disorganization, and impaired cardiac contractility, whereas lamin C overexpression in aged cardiomyocytes preserves normal nuclear morphology and function. Similar age‐dependent reductions in lamin A/C and corresponding nuclear remodeling have been observed in cardiomyocytes of aged mice and nonhuman primates, indicating a conserved aging mechanism across species [23]. Collectively, these findings support the idea that modulating nuclear mechanics may offer a promising strategy to counteract age‐associated functional decline.

#### 5.3.3 Modulating Nuclear Mechanics for Tissue Regeneration

Incorporating principles of nuclear mechanics into tissue‐engineered constructs can significantly improve functional integration and promote construct maturation. Studies of ex vivo cartilage environments show that enzymatic matrix degradation reduces both extracellular matrix stiffness and nuclear envelope stiffness, underscoring mechanical coupling between the extracellular matrix and the nucleus [176]. These findings suggest that tailoring biomaterial matrices with precise mechanical properties could maintain normal nuclear mechanics, thus optimizing cellular function within engineered tissues. Moreover, nuclear mechanical properties have a critical impact on cell migration and matrix remodeling in engineered scaffolds. For instance, transient nuclear softening induced by the histone deacetylase inhibitor Trichostatin A (TSA) has been shown to enhance cell infiltration and improve the spatial distribution of collagen deposition within fibrous scaffolds. Implantation of TSA‐releasing biomaterials into animal models further demonstrated increased endogenous cell infiltration, underscoring the importance of nuclear mechanics for in vivo cellularization of scaffolds [177].

Nuclear stiffness—regulated primarily by chromatin condensation and nuclear lamina composition [178] plays a pivotal role in stem cell differentiation. Increased nuclear stiffness during mesenchymal stem cell differentiation correlates with enhanced lamin A/C expression and greater heterochromatin content. These differentiated cells exhibit heightened mechanosensitivity, such as increased calcium signaling in response to mechanical stimulation, compared to undifferentiated cells. Pharmacological modulation studies indicate that softening the nuclei of differentiated cells reduces mechanosensitivity, whereas stiffening the nuclei of stem cells enhances responsiveness [179]. During the transition of embryonic stem cells before lineage commitment, a temporary reduction in nuclear stiffness accompanied by chromatin decondensation occurs, highlighting the dynamic mechanical regulation [180]. Notably, changes in chromatin organization and nuclear mechanics during stem cell differentiation have been reported to follow distinct trajectories depending on developmental stage and cellular context. For example, in embryonic stem cells, chromatin decondensation, accompanied by nuclear softening, has been observed during the transition as cells prepare for differentiation, which occurs before they initiate lineage commitment [180]. By contrast, a progressive global condensation of chromatin has been reported, with restriction of lineage potential across pluripotent embryonic stem cells, multipotent hematopoietic stem and progenitor cells, and mature hematopoietic cells. Inhibition of the histone methyltransferase G9A, a mediator of H3K9 methylation and heterochromatin formation, delays hematopoietic stem cell differentiation [181]. Additionally, mechanical compression of the nucleus in mesenchymal stem cells increases nuclear membrane curvature, which promotes the nuclear localization of Yes‐associated protein and thereby biases differentiation toward an osteogenic fate. In contrast, lower nuclear curvature favors adipogenic differentiation [182]. These insights suggest that future strategies could precisely modulate nuclear mechanical properties to guide stem cell differentiation, facilitating the generation of specific cell types essential for engineered tissues with tailored functional outcomes.

## 6 Conclusion and Perspectives

This review establishes that nuclear mechanical properties are inherently scale‐dependent. Smaller probes engage the viscous nucleoplasm that permeates the chromatin mesh, whereas larger probes recruit the entropic elasticity of the chromatin network and the structural resistance of the nuclear envelope. This scale dependence is not a measurement artifact but a direct physical consequence of the layered nuclear architecture. The evidence assembled here further shows that mechanical properties at each level, from DNA bendability and chromatin viscoelasticity to lamina stiffness and condensate surface tension, regulate gene expression, chromatin accessibility, nuclear transport, and cell migration. The nucleus is therefore not a passive container but an organelle whose physical state directly influences biological function.

This understanding positions nuclear mechanics as a quantitative variable with direct translational relevance. Nuclear stiffness and deformability serve as label‐free biomarkers that distinguish disease states and inform clinical prognosis. The interplay between nanocarrier deformability and nuclear pore viscoelasticity defines the physical constraints governing nucleus‐targeted delivery. Mechanotherapeutic approaches exploit differences in nuclear stiffness between healthy and diseased cells to achieve selective cytotoxicity. In tissue engineering, matching scaffold mechanics to native nuclear properties promotes cell infiltration and directs differentiation. Across these applications, nuclear mechanics functions as a parameter that can be measured and modulated, rather than simply a descriptor of cellular state.

Realizing the full potential of this framework requires addressing two key gaps. First, most current measurements are performed on isolated cells or 2D substrates, conditions that decouple the nucleus from its native 3D mechanical environment. The resulting parameters may not accurately reflect those operating in tissues. Closing this gap requires in situ mechanotyping technologies, such as advanced Brillouin imaging and functionalized molecular sensors, capable of mapping intranuclear mechanics within 3D tissues and organoids. Second, the mechanical properties of membraneless organelles beyond the nucleolus remain largely uncharacterized. Transcriptional condensates and nuclear speckles concentrate key regulatory machinery, and their rheology likely contributes to the physical regulation of gene expression in ways that are currently undefined.

Addressing these gaps will require coordinated advances. In situ mechanotyping must extend quantitative measurements into physiologically relevant 3D environments. Multiscale computational models must incorporate condensate mechanics and mechanometabolic coupling to connect molecular interactions with organelle‐level behavior. AI‐driven phenotyping must move beyond morphological features to integrate quantitative mechanical parameters, grounding diagnostic scoring in direct physical measurements. Together, these efforts will move nuclear mechanics from a property measured primarily ex vivo to a practical design variable for precision medicine.

## Conflicts of Interest

The authors declare no conflicts of interest.

## Data Availability

The data that support the findings of this study are available on request from the corresponding author. The data are not publicly available due to privacy or ethical restrictions.
